# Valuing the Future and Discounting in Random Environments: A Review

**DOI:** 10.3390/e24040496

**Published:** 2022-04-01

**Authors:** Jaume Masoliver, Miquel Montero, Josep Perelló, J. Doyne Farmer, John Geanakoplos

**Affiliations:** 1 Departament de Física de la Matèria Condensada, Universitat de Barcelona, 08028 Barcelona, Spain; 2 Universitat de Barcelona Institute of Complex Systems (UBICS), 08028 Barcelona, Spain; 3 Institute for New Economic Thinking at the Oxford Martin School, Oxford OX1 3UQ, UK; 4 Mathematical Institute, University of Oxford, Oxford OX2 6GG, UK; 5 Santa Fe Institute, Santa Fe, NM 87501, USA; john.geanakoplos@yale.edu; 6 Department of Economics, Yale University, New Heaven, CT 06511, USA

**Keywords:** discounting, bond pricing, real interest rates, econophysics

## Abstract

We address the process of discounting in random environments, which allows valuation of the future in economic terms. We review several approaches to the problem regarding different well-established stochastic market dynamics in the continuous-time context and include the Feynman–Kac approach. We also review the relation between bond-pricing theory and discounting and introduce both the market price of risk and the risk neutral measure from an intuitive point of view devoid of excessive formalism. We provide the discount for each economic model and discuss their key results. We finally present a summary of our previous empirical studies for several countries on the long-run discount problem.

## 1. Introduction

The introduction around three decades ago of the view and methods of statistical physics into economics and finance signaled the appearance of a new interdisciplinary aspect of physics, which is sometimes called “econophysics” [1,2,3]. The fact that financial prices are random with sudden and uncontrollable ups and downs has been long-known; however, the first step towards a systematic mathematical analysis of price randomness was taken by Bachelier in 1900, who proposed a model for the market dynamics in which the prices follow ordinary Brownian motion [4].

However, Bachelier’s model is not completely satisfactory because, in such a representation, prices can be either positive or negative, contradicting one of the most fundamental tenets of economics, the “principle of limited liability”, which affirms that prices cannot attain negative values. This limitation in Bachelier’s model was remedied more than six decades later by Osborne [5] by assuming the geometric Brownian motion where prices are described by the exponential of the ordinary Brownian motion and, hence, they can never attain negative values.

Let us denote by S(t) a speculative price (or an economic index) at time *t*. In the continuous time framework, the geometric Brownian motion assumes that

(1) $$S\left(t\right)=S_{0}e^{x(t)},$$

where $S_{0}=S\left(t_{0}\right)$ is the price at some initial time $t_{0}$ and x(t), the so-called return, is described by the ordinary Brownian motion, that is to say, by the stochastic differential equation

(2) dx(t)=mdt+σdW(t),

where W(t) is the standard Wiener process with zero mean and unit variance. Note that the return is assumed to be a diffusion process with constant drift *m* and diffusion coefficient σ. In this model, the return is a Gaussian process with the mean and variance given, respectively, by *m* and $\sigma^{2}$. The price is, hence, a log-normal process, and the geometric Brownian motion is also called the log-normal model.

Despite the log-normal model being used in countless financial applications, it has certain limitations [6], which have given rise to several generalizations. One of them assumes that the return is a more complex diffusion process obeying a stochastic equation of the form

(3) dx(t)=f(x,t)dt+g(x,t)dW(t),

which is interpreted in the sense of Itô. In this case, returns, and hence prices, are driven by an external “force” and by multiplicative noise, which, in the most general case, depend explicitly on the time and on the return level. Function f(x,t) drives prices, and function g(x,t) modulates the intensity of the fluctuations around the deterministic motion set by f(x,t). In any case, and regardless of the values taken by x(t), the prices given by Equation (1) are always nonnegative, thus, keeping the principle of limited liability.

Another significant shortcoming of the geometric Brownian motion model is the absence of both “fat tails” and skewness in the distribution of log-prices (i.e., returns). Indeed, empirical distributions of log-prices not only show fat tails—meaning that extreme losses and profits have a higher probability than those of the log-normal model—but also an asymmetric shape in the sense that losses are usually more probable than profits [6]. In order to address these and other problems, intense research has been prompted both in mathematics and physics, which, among others, may involve the use of the Lévy process as driving noise (instead of the Wiener process). One of the most popular alternative financial models—proposed by Mandelbrot [7] and Fama [8] in the early 1960s—is provided by substituting the Wiener process by the Lévy process, which can take into account the appearance of fat tails in the probability distribution of prices, a widely accepted empirical fact [6]. A major inconvenience of the non-Poissonian Lévy jump processes is, however, their lack of finite moments apart from (at most) the first one, which does not seem to be case in empirical data [6]. or models in which the variance $\sigma^{2}$ (or the noise intensity *g*) is a random process, such as in the so-called “stochastic volatility models” [9,10,11].

In economics and finance, one of the most consequential developments is that of “discounting”, which essentially attempts to answer the crucial question of what the price will be in the future. In other words, discounting weighs the future relative to the present. Traditionally, the weighting procedure has been performed through a decreasing exponential. Thus, under a constant interest rate *r*, continuously compounded, a dollar invested today at time t=0 yields $e^{rt}$ dollars at time *t* [12]; hence, one dollar in any future time *t* is worth $e^{-rt}$ today. This statement is true under constant and fixed rates; however, in real life, rates are random, and this uncertainty makes it completely unrealistic to represent rates by constant quantities or even by deterministic functions of time, and, as a consequence, random models for rates must be addressed.

The problem of discounting is widely known in finance where it has been thoroughly studied closely related to bond pricing particularly over short periods of time [13]. Discounting, particularly in the long run, is of importance not only in the context of finance but to many other aspects of the global economy. For instance, we may consider the long-term environmental planning, which is certainly sensitive in relation to climate action. Thus, in an oversimplified way, an environmental problem, which costs *X* to fix at a time *t* is worth an investment of $e^{-rt}X$ today.

This analysis assumes that the interest rate remains constant between today and the distant future *t*. The rate *r* becomes a key magnitude to decide whether it becomes more beneficial to take action today with a significant investment or whether the discount gives negligible value to today’s investment. The choice of discount rate is perhaps the biggest factor influencing the debate on the urgency of the response to global warming as it relates today’s investments with potential climate-related losses in future [14]. No wonder that, in recent years, obtaining a long-run discount rate valid for decades ahead has been the object of intense controversy.

Thus, Nicholas Stern, in an influential report commissioned by the UK government, advocated for a long-run discount of 1.4% [15], which on a 100-year horizon implies a present value of 25% (meaning that the future is worth 25% as much as the present). On the other hand, Willian Nordhaus proposed a discount rate of 4% [16] (implying a present value of 2%) and even the higher value of 6% [17], which implies a present value of 0.3%. Stern has been widely criticized for using such a low rate [18,19,20], and the question is far from being settled.

Economists present a variety of reasons for discounting, particularly for environmental problems in the long run. These reasons include, among others, ethical considerations [21,22], impatience, economic growth [23] and arguments based on the maximization of utility functions that are mostly chosen for mathematical convenience [24], all of them ingrained in a phenomenological expression called the Ramsey formula [25], which constitutes the standard approach to discounting in the economics literature (particularly in the long-run) [14].

From an empirical point of view, any practical economist involved in environmental debates might consider the average of historical interest rates, which occurred in the last 200 hundred years to estimate the forward discount rate (which is 2.7% in the less unstable countries [26]) or take the average of Wall Street forward looking models, with price bonds of maturity as long as 30 years. Unfortunately, due to historical fluctuations of real interest rates, the appropriate rate is considerably below such averages [26].

In econophysics, the problem of discounting, despite its relevance, is virtually unknown. The main purpose of this paper is to offer a survey of the problem devoid of excessive formalism and abstraction as well as to review some of our recent work on the problem [26,27,28,29,30]

## 2. The Process of Discounting—Fundamentals

Let us denote by M=M(t) a given quantity of wealth at time *t*. In economics, the increment of M(t) is assumed to be proportional to the quantity itself and the duration of the variation. For a continuous and instantaneous infinitesimal variation, this can be written as

(4) dM(t)∝M(t)dt.

This starting phenomenological law is built on the empirical observation that the larger M(t), the greater its variation at a given time along with the simpler assumption that such a variation is linear in M(t) and not, for instance, quadratic.

### 2.1. Definitions and General Setting

We define the interest rate as the relative time derivative

(5) $$r\left(t\right)\equiv \frac{1}{M(t)}\frac{dM(t)}{dt}=\frac{d\ln M(t)}{dt},$$

i.e., the rate is the time derivative of the logarithm of wealth. Let us incidentally note that the linearity shown in Equation (4) is equivalent to assume that r(t) is independent of M(t).

In the simplest case, the law (4) represents a direct proportionality, that is to say, r(t) is constant and and from (5), we see that

(6) dM(t)=rM(t)dt,

where *r* is the constant interest rate that has units of 1/(time) (wealth is assumed to be dimensionless). By direct integration, we have the usual exponential law [12]

(7) $$M\left(t\right)=e^{r(t-t_{0})}M\left(t_{0}\right),$$

which connects wealth at some time $t_{0}$, for instance, today (which, without loss of generality, we can take equal to zero) with wealth at some future time $t>t_{0}$.

The growth law (6) appears in many branches of natural and social sciences. Thus, in radioactivity, if N(t) represents the number of active nuclei at time *t*, the usual hypothesis is that this number decreases as

dN(t)=−λN(t)dt.

where λ>0 is the decay constant. Similar considerations apply to many other situations, as in chemical reactions or population dynamics, to name a few.

As we mentioned above, discounting is the procedure of linking wealth at different times. This is done through the discount function defined as

(8) $$\delta \left(t\right)\equiv \frac{M(0)}{M(t)},$$

where M(0) is today’s wealth. In the case of constant rates, we see from Equation (7) that this function is given by the decreasing exponential

(9) $$\delta \left(t\right)=e^{-rt}.$$

The assumption of constant rates is actually unrealistic. A first generalization would be to assume that rates are known functions of time r=r(t). In such a case, the growth law (6) would be given by

(10) dM(t)=r(t)M(t)dt,

which, after integrating, yields

(11) $$\delta \left(t\right)=\exp\left(-\int_{0}^{t}r\left(t^{\prime }\right)dt^{\prime }\right).$$

However, the assumption of rates being given by constants or by deterministic functions of time is unjustified, in particular over long periods of time. Financial interest rates are typically described as random, as the many models for stochastic interest rates appearing in the literature [13,31,32]. In other words, r(t) is a random function of time, and in consequence the discount function δ(t) given by Equation (11) is a stochastic process. The effective discount function is then defined as the average of δ(t),

(12) $$D\left(t\right)=E\left[\exp\left(-\int_{0}^{t}r\left(t^{\prime }\right)dt^{\prime }\right)\right],$$

taken over all possible realizations of r(t). The function r(t) can, in principle, be any random process. However, the most natural and simplifying assumption is that rates are Markovian processes with continuous paths—that is, they are diffusion processes [13]. This approach was proposed after the success of taking an identical approach to model stock prices with log-normal process in 1959 and contrasting with empirical data [5]. The first interest rate model was proposed by O. Vasicek in 1977 [33] and during the same decade when stochastic differential equations became crucial to obtain European option prices. Therefore, rates are solutions to stochastic differential equations of the form

(13) dr=f(r)dt+g(r)dW(t),

where W(t) is the Wiener process and the stochastic differential equation is interpreted in the sense of Itô. We assume that drift f(r) and noise term g(r) do not depend explicitly on time, that is to say, the time dependence is only implicit through r=r(t), which means that the interest rate process is time homogeneous and may be stationary [34]. This is certainly an idealization because real markets do not appear to be time-homogeneous, at least over long periods of time [35].

On the other hand, explicit expressions for f(r) and g(r) should be proposed based on characteristics obtained by actual data, which is observed to have a reversion toward a mean value, and it is thus claimed to attain a stationary regime in contrast with, for instance, stock market price evolution where no stationary behavior is observed. We return to the comparison between models and empirical data in Section 4.

In order to obtain an operative expression for the effective discount function (12), we define the additional random process

(14) $$x\left(t\right)=\int_{0}^{t}r\left(t^{\prime }\right)dt^{\prime }.$$

The interpretation of x(t) is apparent after substituting Equation (5) into Equation (14) and integrating. We find

$$x\left(t\right)=\ln\frac{M(t)}{M(0)}\;\Rightarrow \;M\left(t\right)=M\left(0\right)e^{x(t)},$$

which can be taken as an alternative definition of the accumulated return x(t).

Substituting Equation (14) into Equation (12), we see that the effective discount function can be written as

$$D\left(t\right)=E\left[e^{-x(t)}\right],$$

which implies that, in terms of the probability density function (PDF) $p(x,r,t|r_{0})$ of the bidimensional diffusion process (x(t),r(t)), we can write

(15) $$D\left(t|r_{0}\right)=\int_{-\infty }^{\infty }dr\int_{-\infty }^{\infty }e^{-x}p\left(x,r,t|r_{0}\right)dx,$$

where we have included the dependence on the initial rate, $r_{0}=r\left(0\right)$, in the discount function $D(t|r_{0})$.

From Equations (13) and (14), we see that the bidimensional process (x(t),r(t)) is defined by the following pair of stochastic differential equations

(16) $$\begin{array}{ccc} dx & = & rdt,\\ dr & = & f(r)dt+g(r)dW(t). \end{array}$$

Therefore, the joint density obeys the (forward) Fokker–Planck equation (FPE) [34]

(17) $$\frac{\partial p}{\partial t}=-r\frac{\partial p}{\partial x}-\frac{\partial }{\partial r}\left[f\left(r\right)p\right]+\frac{1}{2}\frac{\partial^{2}}{\partial r^{2}}\left[g^{2}\left(r\right)p\right],$$

with the initial condition

(18) $$p\left(x,r,0|r_{0}\right)=\delta \left(x\right)\delta \left(r-r_{0}\right).$$

After solving the initial-value problem (17)—(18) and obtaining the joint PDF $p(x,r,t|r_{0})$, the discount function follows from Equation (15). There are, however, two different approaches for achieving it. One of them, which is standard in the financial literature, is based on the backward Fokker–Planck equation, and this is called the *Feynman–Kac approach* [13]. A second procedure is based on Fourier analysis [27]. We will explain both approaches next.

### 2.2. The Feynman–Kac Approach

Using this method, one obtains a partial differential for the discount function $D(t|r_{0})$, which is based on the backward Fokker–Planck equation for the joint density $p(x,r,t|r_{0})$. In what follows, we assume that $t_{0}\neq 0$ and denote $x_{0}=x\left(t_{0}\right)$. By definition, $x_{0}=0$ (cf. Equation (14)). However, we temporally keep $x_{0}\neq 0$ and set $x_{0}=0$ at the end of the calculation when needed.

The backward FPE for the PDF $p(x,r,t|x_{0},r_{0},t_{0})$ that corresponds to the bidimensional process (16) is [34]

(19) $$\frac{\partial p}{\partial t_{0}}=-r_{0}\frac{\partial p}{\partial x_{0}}-f\left(r_{0}\right)\frac{\partial p}{\partial r_{0}}-\frac{1}{2}g^{2}\left(r_{0}\right)\frac{\partial^{2}p}{\partial r_{0}^{2}},$$

with the *final condition* as $t_{0}\to t$,

(20) $$p\left(x,r,t|x_{0},r_{0},t\right)=\delta \left(x-x_{0}\right)\delta \left(r-r_{0}\right).$$

Let us observe that the problem (19)—(20) is invariant under translations of both time and $x_{0}$. We thus define the new variables

(21) $$t^{\prime }=t-t_{0},\;x^{\prime }=x-x_{0},$$

so that

$$\frac{\partial p}{\partial t_{0}}=-\frac{\partial p}{\partial t^{\prime }},\;\frac{\partial p}{\partial x_{0}}=-\frac{\partial p}{\partial x^{\prime }},$$

and Equation (19) reads

(22) $$\frac{\partial p}{\partial t^{\prime }}=-r_{0}\frac{\partial p}{\partial x^{\prime }}+f\left(r_{0}\right)\frac{\partial p}{\partial r_{0}}+\frac{1}{2}g^{2}\left(r_{0}\right)\frac{\partial^{2}p}{\partial r_{0}^{2}}.$$

Under this change of variables, we also have

$$p=p\left(x,r,t|x_{0},r_{0},t_{0}\right)=p\left(x,r,t|x-x^{\prime },r_{0},t-t^{\prime }\right)=p\left(x^{\prime },r,t^{\prime }|r_{0}\right),$$

where the last equality comes from the invariance under time and *x* translations, that is,

$$p\left(x,r,t|x_{0},r_{0},t_{0}\right)=p\left(x-x_{0},r,t-t_{0}|r_{0}\right).$$

Consequently, the final condition (20) becomes the initial condition

(23) $$p\left(x^{\prime },r,t^{\prime }=0|r_{0}\right)=\delta \left(x^{\prime }\right)\delta \left(r-r_{0}\right).$$

Having set the backward FPE in the form given by Equation (22), we next obtain the equation satisfied by the effective discount $D(t|r_{0})$. To this end, we multiply Equation (22) by $e^{-x^{\prime }}$ and integrate over $x^{\prime }$ and *r*, we have

(24) $$\begin{array}{ccc} \frac{\partial }{\partial t^{\prime }}\int_{-\infty }^{\infty }dr\int_{-\infty }^{\infty }e^{-x^{\prime }}pdx^{\prime } & = & -r_{0}\int_{-\infty }^{\infty }dr\int_{-\infty }^{\infty }e^{-x^{\prime }}\frac{\partial p}{\partial x^{\prime }}dx^{\prime }\\ & & +\left[f\left(r_{0}\right)\frac{\partial }{\partial r_{0}}+\frac{1}{2}g^{2}\left(r_{0}\right)\frac{\partial^{2}}{\partial r_{0}^{2}}\right]\int_{-\infty }^{\infty }dr\int_{-\infty }^{\infty }e^{-x^{\prime }}pdx^{\prime }. \end{array}$$

From Equation (15), we see that

(25) $$\int_{-\infty }^{\infty }dr\int_{-\infty }^{\infty }e^{-x^{\prime }}p\left(x^{\prime },r,t^{\prime }|r_{0}\right)dx^{\prime }=D\left(t^{\prime }|r_{0}\right).$$

On the other hand, integrating by parts, the first integral on the right hand side of Equation (24) and using (25), we have

(26) $$\int_{-\infty }^{\infty }dr\int_{-\infty }^{\infty }e^{-x^{\prime }}\frac{\partial p}{\partial x^{\prime }}dx^{\prime }=\int_{-\infty }^{\infty }dr\int_{-\infty }^{\infty }e^{-x^{\prime }}p\left(x^{\prime },r,t^{\prime }|r_{0}\right)dx^{\prime }=D\left(t^{\prime }|r_{0}\right),$$

where we have considered the boundary condition (otherwise implicit in the definition of *D* given in Equation (15))

$$\lim_{x^{\prime }\to \pm \infty }\left[e^{-x^{\prime }}p\left(x^{\prime },r,t^{\prime }|r_{0}\right)\right]=0.$$

Substituting Equations (25) and (26) into Equation (24) and setting $t_{0}=0$, which implies $t^{\prime }=t$ [cf. Equation (21)], we finally obtain

(27) $$\frac{\partial D}{\partial t}=-r_{0}D+f\left(r_{0}\right)\frac{\partial D}{\partial r_{0}}+\frac{1}{2}g^{2}\left(r_{0}\right)\frac{\partial^{2}D}{\partial r_{0}^{2}},$$

with the initial condition (cf. Equations (23) and (25))

(28) $$D(0|r_{0})=1.$$

The method for obtaining the discount function $D(t|r_{0})$ by solving the initial-value problem (27)—(28) is called the *Feynman–Kac approach*, and Equation is (27) the *Feynman–Kac equation*. In some applications (see, for instance, Section 3), it is convenient to consider $t_{0}\neq 0$ so that $t^{\prime }=t-t_{0}\neq t$. In these cases, it is appropriate to denote $D=D(t|r_{0},t_{0})$ and the Feynman–Kac Equation (27) reads

(29) $$\frac{\partial D}{\partial t_{0}}=r_{0}D-f\left(r_{0}\right)\frac{\partial D}{\partial r_{0}}-\frac{1}{2}g^{2}\left(r_{0}\right)\frac{\partial^{2}D}{\partial r_{0}^{2}},$$

with the final condition $D(t|r_{0},t)=1$.

### 2.3. The Fourier Transform Approach

An alternative method for obtaining the discount function is based on the joint characteristic function—that is, on the Fourier transform of the joint density,

(30) $$\tilde{p}\left(\omega_{1},\omega_{2},t|r_{0}\right)=\int_{-\infty }^{\infty }e^{-i\omega_{2}r}dr\int_{-\infty }^{\infty }e^{-i\omega_{1}x}p\left(x,r,t|r_{0}\right)dx.$$

One of the chief advantages of working with the characteristic function is that obtaining the effective discount is straightforward. Indeed, comparison of Equation (15),

$$D\left(t|r_{0}\right)=\int_{-\infty }^{\infty }dr\int_{-\infty }^{\infty }e^{-x}p\left(x,r,t|r_{0}\right)dx,$$

with Equation (30) shows that

(31) $$D\left(t|r_{0}\right)=\tilde{p}\left(\omega_{1}=-i,\omega_{2}=0,t|r_{0}\right).$$

Therefore, in order to obtain the discount function, we only need to know the joint characteristic function of the bidimensional process (x,r). The procedure is quite advantageous in linear cases. In a forthcoming section, we will apply this approach to some standard models of interest rates.

### 2.4. Adding Risk Aversion

As we will see in the next section, the process of discounting just described is very closely related to an important problem in finance called bond pricing. In the context of bond pricing, there can be two kinds of investors. For one hand, if investors are *risk neutral*, then bond prices can be modeled based on the data generating measure *p*, which is the solution of the Fokker–Planck Equation (17) with the initial condition (18). This is sometimes called *the Local Expectation Hypothesis* (LEH) [36,37]. Nonetheless, a more general assumption is that investors are sensitive to risk.

In such a case, bonds are somewhat more accurately priced using an artificial density $p^{*}$ usually called a *risk-neutral (or risk-correcting) probability measure*. Both magnitudes, the data generating measure *p* and the risk-neutral measure $p^{*}$, are related through a quantity that is denoted by q(r,t) and called *market price of risk*, which, as described in the next section, is the extra return per unit of risk that investors demand to bear risk. This additional return is thus determined by a function q=q(r,t) that, in its most general form, may depend on the rate *r* and current time *t*, although the most usual assumption is that q=q(r) only depends on the rate [33]. Following a standard procedure for bond pricing [33,38], which we will present in Section 3, one takes risk into account by replacing the drift f(r) by $f^{*}\left(r\right)$,

$$f\left(r\right)\to f^{*}\left(r\right),$$

where

(32) $$f^{*}\left(r\right)=f\left(r\right)+g\left(r\right)q\left(r\right),$$

and q(r)≥0 is the market price of risk. The form of q(r) is, in principle, unknown and has to be conjectured. The simplest and most common assumption is that q(r)=q is constant, in such a case, the value of *q* may be more easily estimated from empirical data. Now, the risk-neutral measure $p^{*}\left(x,r,t|r_{0}\right)$ is given by the Fokker–Planck Equation (17) with f(r) replaced by $f^{*}\left(r\right)$; that is,

(33) $$\frac{\partial p^{*}}{\partial t}=-r\frac{\partial p^{*}}{\partial x}-\frac{\partial }{\partial r}\left[\left[f(r)+g(r)q(r)\right]p^{*}\right]+\frac{1}{2}\frac{\partial^{2}}{\partial r^{2}}\left[g^{2}\left(r\right)p^{*}\right],$$

with the initial condition given by

(34) $$p^{*}\left(x,r,0|r_{0}\right)=\delta \left(x\right)\delta \left(r-r_{0}\right).$$

In an analogous way, the discount function adjusted for risk will now be given by the Feynman–Kac Equation (27) with f(r) replaced by $f^{*}\left(r\right)$. Or, using the Fourier method, the discount function will be given in terms of the risk-neutral characteristic function, ${\tilde{p}}^{*}\left(\omega_{1},\omega_{2},t|r_{0}\right)$, by (cf. Equation (31))

(35) $$D\left(t|r_{0}\right)={\tilde{p}}^{*}\left(\omega_{1}=-i,\omega_{2}=0,t|r_{0}\right).$$

## 3. Pricing Bonds—The Term Structure of Interest Rates

Pricing bonds is a traditional objective in finance and intimately related to the problem of discounting. It constitutes a vast subject with countless studies, many of them rather abstract, which have appeared in the mathematical finance literature over the last decades. We present a short and intentionally simple, yet rigorous, introduction to the subject devoid as much as possible of technicalities and mathematical subtleties and refer the interested reader to more specialized works for further information [13].

A bond is a financial instrument that one purchases now and that provides a payment in the future. From a more technical point of view, we say that a (discount) bond is a default-free claim on a specified sum of money to be delivered at a given future date called the maturity time. Such claims are bought and issued by investors. Let us denote by $B(t_{0},t)$ the price at time $t_{0}$ of a discount bond maturing at time $t\geq t_{0}$, with unit maturity value,

B(t,t)=1.

Let us incidentally note that, if the final maturity price is not 1 (say, B(t,t)=β) then the price of the bond at $t_{0}$ would be $\beta B(t_{0},t)$.

Bonds are classified according to the *time interval to maturity* τ defined as

$$\tau =t-t_{0}.$$

Thus, if τ=10 years, we talk about a 10-year bond that is traded at $t_{0}$ (for instance, today) with price $B(t_{0},t_{0}+10)$ and that, after 10 years, has unit value. Similarly for a 3-year bond, 3-month bond, etc.

The central question is to know the *backward evolution* of the bond price, from unit maturity to the initial purchasing price $B(t_{0},t)$. Note that the problem is virtually identical to the problem of discounting discussed in Sect. II, with the sole difference that, in discounting, we look for the forward evolution from a known initial value to an unknown final value, while, in bond pricing, the situation is reversed, since we know the final value but not the initial one.

In order to proceed further, we define the *instantaneous rate of return* $b(t_{0},t)$ (also called *forward rate*) as the relative time variation of the bond price (compare with Equation (5))

(36) $$b\left(t_{0},t\right)\equiv \frac{1}{B(t_{0},t)}\frac{\partial B(t_{0},t)}{\partial t_{0}}=\frac{\partial \ln B(t_{0},t)}{\partial t_{0}}.$$

The knowledge of the forward rate $b(t_{0},t)$ allows us to relate the initial price $B(t_{0},t)$ and the maturing price B(t,t)=1. Indeed, the integration of the above equation directly leads to

(37) $$B\left(t_{0},t\right)=\exp\left[-\int_{t_{0}}^{t}b\left(t_{0}^{\prime },t\right)dt_{0}^{\prime }\right].$$

The close analogy between bond pricing and discounting is now apparent. Indeed, the comparison of Equation (37) with Equation (11) shows that $B(t_{0},t)$ is the equivalent of the discount function δ(t) and that the forward rate $b(t_{0},t)$ is the equivalent of the discount rate r(t). However, in what follows, we will use the notation r(t) not for the forward rate $b(t_{0},t)$ but for the so-called spot rate (also called nominal rate), which we define in Equation (39).

Another quantity of interest is the *yield to maturity* $y(t_{0},\tau )$ defined by

(38) $$y\left(t_{0},\tau \right)\equiv -\frac{1}{\tau }\ln B\left(t_{0},t_{0}+\tau \right)\;\Rightarrow \;B\left(t_{0},t_{0}+\tau \right)=e^{-\tau y(t_{0},\tau )}.$$

From (37), we see that

$$y\left(t_{0},\tau \right)=\frac{1}{\tau }\int_{t_{0}}^{t_{0}+\tau }b\left(t_{0}^{\prime },t\right)dt_{0}^{\prime },$$

that is to say, the yield is the time average of the forward rate over the maturity period τ.

A final quantity is needed, the *spot or nominal rate*, which is defined as the limit of the yield when the maturity tends to 0. In dealing with bonds, one sometimes uses, for the nominal rate, the notation $n(t_{0})$ instead of $r(t_{0})$—the later reserved for real interest rates, which can be negative due to inflation (see Section 3).

(39) $$r\left(t_{0}\right)\equiv \lim_{\tau \to 0}y\left(t_{0},\tau \right)=\lim_{\tau \to 0}\left[\frac{1}{\tau }\int_{t_{0}}^{t_{0}+\tau }b\left(t_{0}^{\prime },t\right)dt_{0}^{\prime }\right].$$

Solving the indeterminacy by expanding the integral in powers of τ, we see that the spot rate is given in terms of the forward rate by

(40) $$r\left(t_{0}\right)=b\left(t_{0},t_{0}\right).$$

In other words, the spot rate is the instantaneous forward rate.

Let us finally note that a loan of amount *M* subscribed at time $t_{0}$ with an interest rate $r(t_{0})$ (the spot rate) will, at time $t_{0}+dt_{0}$, increase in value to M+dM, where

(41) $$dM=r\left(t_{0}\right)Mdt_{0}.$$

Indeed, at any time $t_{0}$, the value of the spot rate $r(t_{0})$ is the instantaneous increase of the loan value, that is, $r\left(t_{0}\right)=d\ln M\left(t_{0}\right)/dt_{0}$ (compare with Equation (36)). All of this clearly heightens the close similarities with discounting mentioned above.

However, subsequent values of the spot rate are not necessarily certain. We will see next, the consequences of this fact on the time evolution of the bond price $B(t_{0},t)$.

### 3.1. Dynamics of the Bond Price

Suppose the spot rate $r(t_{0})$ is not deterministic but random. In such a case, and analogously to discounting, the usual assumption is that $r_{0}=r\left(t_{0}\right)$ is a Markovian random process with continuous trajectories; that is, a diffusion process obeying a stochastic differential equation of the form

(42) $$dr_{0}=f\left(r_{0}\right)dt_{0}+g\left(r_{0}\right)dW\left(t_{0}\right),$$

where $W(t_{0})$ is the standard Wiener process. We assumed that the drift and the noise intensity are independent of time, thus, the time dependence of these coefficients is implicit through $r_{0}=r\left(t_{0}\right)$. We know that this implies invariance under time translations, and we can set $t_{0}=0$ when needed without loss of generality.

We will now obtain the time evolution of the bond price $B(t_{0},t)$ from the purchasing time $t_{0}$ to maturity *t*, and to this end, we follow Oldrich Vasicek [33]. Let us first observe that the most natural hypothesis consists in assuming that the bond price *B* is a function of the initial spot rate $r_{0}=r\left(t_{0}\right)$ and write

(43) $$B=B\left[t_{0},t|r\left(t_{0}\right)\right].$$

In this way, $B(t_{0},t|r_{0})$ represents the price of a bond issued at time $t_{0}$ and maturing at time *t*, given that the initial interest rate is $r_{0}=r\left(t_{0}\right)$. The infinitesimal variation of the bond price is then defined by

$$dB=B[t_{0}+dt_{0},t|r\left(t_{0}+dt_{0}\right)]-B\left[t_{0},t|r\left(t_{0}\right)\right].$$

We expand in Taylor series up to second order

$$\begin{array}{ccc} B[t_{0}+dt_{0},t|r(t_{0} & + & dt_{0})]=B\left[t_{0},t|r\left(t_{0}\right)\right]+\frac{\partial B}{\partial t_{0}}dt_{0}+\frac{\partial B}{\partial r_{0}}dr_{0}\\ & + & \frac{1}{2}\left[\frac{\partial^{2}B}{\partial t_{0}^{2}}dt_{0}^{2}+\frac{\partial^{2}B}{\partial r_{0}^{2}}dr_{0}^{2}+2\frac{\partial^{2}B}{\partial t_{0}\partial r_{0}}dt_{0}dr_{0}\right]+\cdots . \end{array}$$

Substituting for Equation (42) and taking into account that $dW\left(t_{0}\right)=O\left(dt_{0}^{1/2}\right)$ [34,39], we write

$$dB=\left[\frac{\partial B}{\partial t_{0}}+f\left(r_{0}\right)\frac{\partial B}{\partial r_{0}}\right]dt_{0}+g\left(r_{0}\right)\frac{\partial B}{\partial r_{0}}dW\left(t_{0}\right)+\frac{1}{2}g^{2}\left(r_{0}\right)\frac{\partial^{2}B}{\partial r_{0}^{2}}{\left[dW(t_{0})\right]}^{2}+O\left(dt_{0}^{3/2}\right).$$

However, ${\left[dW(t_{0})\right]}^{2}=dt_{0}$ (in mean square sense) [34,39], and, up to the first order in $dt_{0}$, we obtain

(44) $$dB=\left[\frac{\partial B}{\partial t_{0}}+f\left(r_{0}\right)\frac{\partial B}{\partial r_{0}}+\frac{1}{2}g^{2}\left(r_{0}\right)\frac{\partial^{2}B}{\partial r_{0}^{2}}\right]dt_{0}+g\left(r_{0}\right)\frac{\partial B}{\partial r_{0}}dW\left(t_{0}\right).$$

Defining

(45) $$\mu \left(t_{0},t|r_{0}\right)\equiv \frac{1}{B}\left[\frac{\partial B}{\partial t_{0}}+f\left(r_{0}\right)\frac{\partial B}{\partial r_{0}}+\frac{1}{2}g^{2}\left(r_{0}\right)\frac{\partial^{2}B}{\partial r_{0}^{2}}\right],$$

and

(46) $$\sigma \left(t_{0},t|r_{0}\right)\equiv -\frac{1}{B}g\left(r_{0}\right)\frac{\partial B}{\partial r_{0}},$$

we see from (44) that the bond price satisfies the stochastic differential equation

(47) $$\frac{dB}{B}=\mu \left(t_{0},t|r_{0}\right)dt_{0}-\sigma \left(t_{0},t|r_{0}\right)dW\left(t_{0}\right),$$

showing that the bond price is also a diffusion process.

Averaging Equation (47) and recalling that $E[dW\left(t_{0}\right)]=0$, we see that

$$\mu \left(t_{0},t|r_{0}\right)=E\left[\frac{1}{B}\frac{dB}{dt_{0}}\right],$$

which proves that $\mu (t_{0},t|r_{0})$ is the average of the instantaneous rate of return [cf. Equation (36)] at time $t_{0}$ on a bond with maturing date *t*, given that the current spot rate is $r_{0}$. In an analogous way, one can easily show that $\sigma^{2}\left(t_{0},t|r_{0}\right)$ is the variance [33].

We therefore see from the above development that the bond price is a random quantity but with a final fixed price (the maturity price). The question is: what is the (initial) price that an investor has to buy (or sell) a bond at time $t_{0}$ maturing at time $t=t_{0}+\tau$ with the current spot rate $r_{0}$? One possible answer would be proceeding as in discounting to take the average over all possible realizations of the bond price. However, this procedure implies that the expected rate of return of a bond is invariant under risk variation—that is, under changes of the variance $\sigma^{2}\left(t_{0},t|r_{0}\right)$—a fact that investors always have in mind.

We explain next a procedure resulting in a deterministic bond price, which takes into account the risk aversion of investors (in practice this is only true to some extend because the mathematical procedure assumes that the market is driven by Gaussian white noise—that is, the Wiener process, which is an idealized noise presenting, among other shortcomings, no fat tails, a key characteristic of real markets [6]).

### 3.2. The Market Price of Risk

Consider an investor who, at time $t_{0}$, sells an amount $M_{1}$ of a bond maturing at time $t_{1}$ and, at the same time, buys an amount $M_{2}$ of another bond with a different maturing date $t_{2}$. The total worth of the *portfolio*, thus, constructed is $M=M_{2}-M_{1}$. Note that each amount $M_{i}$ (i=1,2) is a multiple of the bond price $B(t_{0},t_{i}|r_{0})$ (i=1,2) and, hence, they also obey the stochastic differential Equation (47). That is,

$$\frac{dM_{i}}{M_{i}}=\mu \left(t_{0},t_{i}|r_{0}\right)dt_{0}-\sigma \left(t_{0},t_{i}|r_{0}\right)dW\left(t_{0}\right).$$

As a consequence, the infinitesimal variation $dM=dM_{2}-dM_{1}$ of the worth of the portfolio changes over time according to

(48) $$\begin{array}{ccc} dM & = & \left[\mu \left(t_{0},t_{2}|r_{0}\right)M_{2}-\mu \left(t_{0},t_{1}|r_{0}\right)M_{1}\right]dt_{0}\\ & & -\left[\sigma \left(t_{0},t_{2}|r_{0}\right)M_{2}-\sigma \left(t_{0},t_{1}|r_{0}\right)M_{1}\right]dW\left(t_{0}\right). \end{array}$$

Suppose we choose the amounts $M_{1}$ and $M_{2}$ such that

(49) $$M_{1}=\frac{M}{\sigma_{1}-\sigma_{2}}\sigma_{2},\;M_{2}=\frac{M}{\sigma_{1}-\sigma_{2}}\sigma_{1},$$

where $M=M_{2}-M_{1}$ and $\sigma_{i}=\sigma \left(t_{0},t_{i}|r_{0}\right)$ (i=1,2). Hence $M_{1}$ is proportional to $\sigma_{2}$, while $M_{2}$ is proportional to $\sigma_{1}$. With this choice, we have

$$\sigma_{2}M_{2}-\sigma_{1}M_{1}=\sigma_{2}\frac{\sigma_{1}M}{\sigma_{1}-\sigma_{2}}-\sigma_{1}\frac{\sigma_{2}M}{\sigma_{1}-\sigma_{2}}=0,$$

and the random term in Equation (48) vanishes. This renders the portfolio composed of such amounts of the two bonds instantaneously riskless:

(50) $$dM=\frac{M}{\sigma_{1}-\sigma_{2}}\left(\mu_{2}\sigma_{1}-\mu_{1}\sigma_{2}\right)dt_{0},$$

where $\mu_{i}=\mu \left(t_{0},t_{i}|r_{0}\right)$. The rate of return $r_{M}$ of this portfolio is

$$r_{M}\equiv \frac{1}{M}\frac{dM}{dt_{0}}=\frac{\mu_{2}\sigma_{1}-\mu_{1}\sigma_{2}}{\sigma_{1}-\sigma_{2}}.$$

In order to avoid *arbitrage opportunities*—that is, making profits without taking any risk—the rate $r_{M}$ must be equal to the spot rate $r_{0}$. If not, the portfolio can be purchased by taking funds borrowed at the spot rate, or otherwise sold and the profits lent out to accomplish a riskless arbitrage [33]. Therefore (compare also Equation (41) with Equation (50))

$$r_{0}=\frac{\mu_{2}\sigma_{1}-\mu_{1}\sigma_{2}}{\sigma_{1}-\sigma_{2}}.$$

Rearranging terms, we find $\left(\mu_{1}-r_{0}\right)/\sigma_{1}=\left(\mu_{2}-r_{0}\right)/\sigma_{2}$, so that

$$\frac{\mu \left(t_{0},t_{1}|r_{0}\right)-r_{0}}{\sigma (t_{0},t_{1}|r_{0})}=\frac{\mu \left(t_{0},t_{2}|r_{0}\right)-r_{0}}{\sigma (t_{0},t_{2}|r_{0})}.$$

This equation is valid for arbitrary maturities $t_{1},t_{2},\ldots$, it then follows that the ratio $\left[\mu \left(t_{0},t|r_{0}\right)-r_{0}\right]/\sigma \left(t_{0},t|r_{0}\right)$*must be independent of the maturity timet*.

Let us denote by $q(t_{0}|r_{0})$ the common value of such a ratio for a bond of any maturity date, given that the current spot rate (at time $t_{0}$) is $r_{0}$,

(51) $$q\left(t_{0}|r_{0}\right)\equiv \frac{\mu \left(t_{0},t|r_{0}\right)-r_{0}}{\sigma (t_{0},t|r_{0})},\;\left(t\geq t_{0}\right).$$

The quantity $q(t_{0}|r_{0})$ is called the *market price of risk*, as it gives the variation of the expected rate of return on a bond (specified by the *risk premium*$\mu -r_{0}$) per an additional unit risk (specified by the standard deviation σ). The market price of risk $q(t_{0}|r_{0})$ is the so-called Sharpe ratio [40] of the excess return $\mu -r_{0}$.

Note that, if q=0, the spot rate $r_{0}=r\left(t_{0}\right)$ and the average rate of return μ coincide.

$$\mu \left(t_{0},t|r_{0}\right)=r\left(t_{0}\right)$$

$\left(t=t_{0}+\tau \right)$ meaning that the expected instantaneous rates of return on bonds are the same for all maturities.

### 3.3. The Term Structure Equation and the Risk-Neutral Measure

The introduction of the market price of risk implies a non-random bond price $B=B(t_{0},t|r_{0})$, which, in turn, allows a deterministic equation for *B*. In effect, rewriting Equation (51) as

$$\mu \left(t_{0},t|r_{0}\right)-r_{0}=\sigma \left(t_{0},t|r_{0}\right)q\left(t_{0}|r_{0}\right),$$

and substituting μ and σ for their definitions given in Equations (45) and (46), we have

$$\frac{1}{B}\left[\frac{\partial B}{\partial t_{0}}+f\left(r_{0}\right)\frac{\partial B}{\partial r_{0}}+\frac{1}{2}g^{2}\left(r_{0}\right)\frac{\partial^{2}B}{\partial r_{0}^{2}}\right]-r_{0}=-q\left(t_{0}|r_{0}\right)\frac{1}{B}g\left(r_{0}\right)\frac{\partial B}{\partial r_{0}},$$

which, after rearranging terms, yields

(52) $$\frac{\partial B}{\partial t_{0}}=r_{0}B-\left[f\left(r_{0}\right)+g\left(r_{0}\right)q\left(t_{0}|r_{0}\right)\right]\frac{\partial B}{\partial r_{0}}-\frac{1}{2}g^{2}\left(r_{0}\right)\frac{\partial B}{\partial r_{0}^{2}}.$$

This equation, called the term structure equation, is a partial differential equation for $B(t_{0},t|r_{0})$, that is obtained once we know the random character of the spot rate process r(t) (through its drift *f* and noise intensity *g*) and once the market price of risk $q(t_{0}|r_{0})$ is specified. Bond prices are thus obtained after solving the deterministic Equation (52) with the final condition:

(53) $$B(t,t|r_{0})=1.$$

Let us observe that the term structure Equation (52) for the bond price *B* is identical to the Feynman–Kac Equation (27) for the discount function *D* as long as we make the following change of drift

(54) $$f\left(r_{0}\right)⟶f\left(r_{0}\right)+g\left(r_{0}\right)q\left(t_{0}|r_{0}\right).$$

On the other hand, as we have seen in Section 2, the solution of the Feynman–Kac Equation (27) for the discount function $D(t|r_{0})$ is written as the average (cf. Equation (15))

$$D\left(t|r_{0},t_{0}\right)=\int_{-\infty }^{\infty }dr\int_{-\infty }^{\infty }e^{-x}p\left(x,r,t|r_{0},t_{0}\right)dx,$$

where $p(x,r,t|r_{0},t_{0})$ is the probability density function of the bidimensional diffusion process (x(t),r(t)) defined by Equation (16),

dx=rdt,dr=f(r)dt+g(r)dW(t).

Now the analogy between the term structure Equation (52) and the Feynman–Kac Equation (29) suggests that we can write the bond price $B(t_{0},t|r_{0})$ as an average over the different realizations of the spot rate $r(t_{0})$. However, this averaging procedure is taken using a modified PDF called the *risk-free measure*. Thus, it can be proven in a more rigorous way that [32,33]

(55) $$B\left(t_{0},t|r_{0}\right)=\int_{-\infty }^{\infty }dr\int_{-\infty }^{\infty }e^{-x}p^{*}\left(x,r,t|r_{0},t_{0}\right)dx,$$

where $p^{*}\left(x,r,t|r_{0},t_{0}\right)$ is the risk-free measure that is the PDF of the bidimensional process $\left(x\left(t_{0}\right),r\left(t_{0}\right)\right)$ defined by the following pair of stochastic differential equations that include the market price of risk (see Equation (54)):

(56) $$\begin{array}{ccc} dx & = & rdt,\\ dr & = & [f(r)+g(r)q(t|r)]dt+g(r)dW(t). \end{array}$$

That is, $p^{*}$ is the solution to the FPE

(57) $$\frac{\partial p^{*}}{\partial t}=-r\frac{\partial p^{*}}{\partial x}-\frac{\partial }{\partial r}\left[\left[f(r)+g(r)q(t|r)\right]p^{*}\right]+\frac{1}{2}\frac{\partial^{2}}{\partial r^{2}}\left[g^{2}\left(r\right)p^{*}\right],$$

with the initial condition

(58) $$p^{*}\left(x,r,t_{0}|r_{0},t_{0}\right)=\delta \left(x\right)\delta \left(r-r_{0}\right).$$

Since, as we have shown in Section 2.2, the Feynman–Kac approach to discounting is equivalent to the Fourier method described in Section 2.3, we can apply the latter to directly obtain the bond price knowing only the risk neutral PDF, without having to solve the Feynman–Kac Equation (52) with condition (23). Indeed, the characteristic function of the risk neutral density $p^{*}$ is the joint Fourier transform

$${\tilde{p}}^{*}\left(\omega_{1},\omega_{2},t|r_{0},t_{0}\right)=\int_{-\infty }^{\infty }e^{-i\omega_{2}r}dr\int_{-\infty }^{\infty }e^{-i\omega_{1}x}p^{*}\left(x,r,t|r_{0},t_{0}\right)dx,$$

which, after comparing with Equation (55), yields

(59) $$B\left(t_{0},t|r_{0}\right)={\tilde{p}}^{*}\left(\omega_{1}=-i,\omega_{2}=0,t|r_{0},t_{0}\right).$$

Finally, once we know the bond price, the yield to maturity $y(t_{0},\tau |r_{0})$ (also called *the term structure of interest rates*) is readily evaluated from Equation (38):

(60) $$y\left(t_{0},\tau |r_{0}\right)=-\frac{1}{\tau }\ln B\left(t_{0},t_{0}+\tau |r_{0}\right).$$

The graphic representations of $y(t_{0},\tau |r_{0})$ as a function of $t_{0}$ and for different values of the maturity interval τ are called *yield curves* and are of prime importance for practitioners.

## 4. Standard Models

Throughout the above development, it is clear that, in order to proceed further in the discounting process (as well as in pricing bonds), we need to identify the specific diffusion process chosen for modeling rates. Such a choice is mostly based on the analysis of empirical data [26,29]. Clearly, in any proposed market model, there are idealizations, otherwise a complete treatment of the problem would be very problematic, not to say impossible, not only analytically but computationally as well.

In addition to assuming a diffusive behavior for the market, the first of such idealizations is supposing that the market is stationary, i.e., the structural conditions of the market do not change over time. However, and in particular after the 1980s, market circumstances have largely changed due to a great increase of transaction volumes along with transparency and, to a lesser extend, changes in investor perspectives. In this review, we only address stationary models, although we are working on new models dealing with some non-stationary features of the market [29,35], and we refer the interested reader to these works for further information.

On the other hand, there is a property of the market that appears to be well founded on empirical grounds. This is the property of *mean reversion* meaning that prices tend to return to some fundamental value, called the *normal level*, which is typically identified as the long-time (i.e., stationary) mean value. The simplest method of introducing this feature in the diffusion market model is to assume a linear drift of the form f(r)=−α(r−m), where α>0 is the strength of the reversion to the normal level, identified by *m*.

In such a case, the drift acts like a linear restoring force driving r(t) towards *m* as time increases. Despite that the introduction of mean reversion might create some arbitrage opportunities, the property of mean reversion is widely accepted in the literature [6], and we previously discussed this issue in the context of option pricing when considering the Ornstein–Uhlenbeck model [41].

### 4.1. Bonds and Real Rates

Before proceeding with the introduction of some standard models for the market evolution, we briefly explain the link between bonds and (real) interest rates.

Financial economists have developed a large number of models of interest rate processes to enable them to price bonds and other cash flows. In these models, interest rates are described by positive random processes since financial interest rates rarely take negative values. Although the models could be, in principle, extended to arbitrary horizons, they have only been studied carefully over time horizons of up to 30 years, since bonds are seldom issued for periods longer than this.

On the other hand, environmental economists are interested in the real behavior of the economic growth over longer horizons, in contrast to financial economists who are typically more interested in nominal rates over shorter periods of time. The behavior of real and nominal rates usually differ as, due to inflation, real rates can take on negative values. In this way, real rates r(t) are generally defined by the so-called Fisher procedure:

(61) r(t)=n(t)−i(t),

where i(t) is the inflation rate that is usually generated from consumer price indexes as we will explain in the next section. The quantity n(t) represents nominal rates, which are typically constructed out of government bonds and are usually positive (even though, in recent years, nominal rates have taken slightly negative values). In order to explain the close relationship between nominal rates and bonds, let us first recall that nominal rates were called spot rates in the previous section on bond pricing where we used the notation r(t) instead of n(t). We thus define the spot (i.e., nominal) rate as (see Equations (39) and (40))

(62) $$n\left(t\right)\equiv \lim_{\tau \to 0}\left[\frac{1}{\tau }\int_{t}^{t+\tau }b\left(t^{\prime },t\right)dt^{\prime }\right]=b\left(t,t\right),$$

where $b(t^{\prime },t)$ is the forward rate for bonds defined in Equation (36) (see also Equation (37)), that is

$$b\left(t^{\prime },t\right)=\frac{\partial \ln B(t^{\prime },t)}{\partial t^{\prime }}\;\Rightarrow \;B\left(t^{\prime },t\right)=\exp\left[-\int_{t^{\prime }}^{t}b\left(t^{\prime \prime },t\right)dt^{\prime \prime }\right]$$

where $B(t^{\prime },t)$ is the price at time $t^{\prime }$ of a (government) bond maturing at time $t\geq t^{\prime }$. Let us recall the definition of the yield to maturity y(t,τ) given in Equation (38),

$$y\left(t,\tau \right)\equiv -\frac{1}{\tau }\ln B\left(t,t+\tau \right)\;\Rightarrow \;B\left(t,t+\tau \right)=e^{-\tau y(t,\tau )}$$

(τ≥0), so that

$$y\left(t,\tau \right)=\frac{1}{\tau }\int_{t}^{t+\tau }b\left(t^{\prime },t\right)dt^{\prime }$$

and comparing with Equation (62), we see that, in terms of the yield nominal rates, n(t) can be defined as

(63) $$n\left(t\right)=\lim_{\tau \to 0}y\left(t,\tau \right).$$

Thus, for empirical analysis, the yield can be used as an estimator of the nominal rates:

(64) n(t)∼y(t,τ),

and the accuracy of such an estimator increases as τ→0. We will return to this discussion in the next section.

In this way, taking nominal rates corrected by inflation as a proxy of economic growth, we recently demonstrated [26,29,30] through a detailed empirical study on many countries that real interest rates are negative around 25% of the time (see next section). To understand how discounting depends on the random process used to characterize interest rates, we focused on three different models and obtained exact analytical expressions for the discount function [27]. The three models describe to, varying degrees, a number of relevant characteristics observed in the data, while being simple enough to allow for complete analytical treatment. The main results are summarized in Table 1.

The first model is based on the Ornstein–Uhlenbeck (OU) process—also called the Vasicek model in the financial literature [13]—which allows for negative rates and is, therefore, suitable for pricing environmental problems. The model has a stationary probability distribution and exhibits reversion to the mean, which means that the process tends to return to its average stationary value. We will review this model below.

The second and third models that we considered are given by the Feller and log-normal processes, respectively. For these processes, the rates cannot be negative. The Feller process—also known as the Cox–Ingersoll–Ross (CIR) model [42]—has reversion to the mean and stationary probability distribution.

This is one of the most popular models in finance [13], and we recently reviewed the main properties of the Feller process in previous works [27,43]. A third model, also implying positive rates, is the log-normal process (occasionally called the Dotham model in the financial literature [44]). The model does not have reversion to the mean nor a stationary distribution. Despite these shortcomings, the log-normal process has also been used in the financial literature mainly because it is positive and allows for analytical treatment [13]. We refer the interested reader to our previous work [27] for details on this model.

As remarked in the introduction, we are primarily interested in valuing the far future for environmental problems rather than the short time discount of finance, the latter implying positive interest rates, while the former involves positive as well as negative rates. For this reason, we next review in more detail the Vasicek model allowing for both positive and negative rates than the CIR and log-normal models of which we only present a sketched review.

### 4.2. The Vasicek (Ornstein–Uhlenbeck) Model

In this model, the rates are described by the Ornstein–Uhlenbeck process [33], which is a diffusion model with linear drift and constant noise intensity:

(65) dr(t)=−α[r(t)−m]+kdW(t),

where r(t) is the rate and W(t) is the Wiener process. The parameter *m* (the normal level) is the mean value to which rates revert, k>0 is the amplitude of fluctuations, and α>0 is the strength of the reversion to the mean. These parameters have to be estimated from empirical data.

In this case, the Fokker–Planck equation for the joint density $p(x,r,t|r_{0})$ of the bidimensional process (x(t),r(t)), given by Equation (17), reads

(66) $$\frac{\partial p}{\partial t}=-r\frac{\partial p}{\partial x}+\alpha \frac{\partial }{\partial r}\left[\left(r-m\right)p\right]+\frac{1}{2}k^{2}\frac{\partial^{2}p}{\partial r^{2}},$$

with the initial condition

(67) $$p\left(x,r,0|r_{0}\right)=\delta \left(x\right)\delta \left(r-r_{0}\right).$$

The joint Fourier transform of these equations results in a simpler initial-value problem for the joint characteristic function $\tilde{p}\left(\omega_{1},\omega_{2},t|r_{0}\right)$, which can be readily solved to yield the Gaussian density [27]

(68) $$\tilde{p}\left(\omega_{1},\omega_{2},t|r_{0}\right)=\exp\left\{-A\left(\omega_{1},t\right)\omega_{2}^{2}-B\left(\omega_{1},t|r_{0}\right)\omega_{2}-C\left(\omega_{1},t|r_{0}\right)\right\},$$

where $A(\omega_{1},t)$, $B(\omega_{1},t)$ and $C(\omega_{1},t)$ are given by [27]

(69) $$A\left(\omega_{1},t\right)=\frac{k^{2}}{4\alpha }\left(1-e^{-2\alpha t}\right),$$

(70) $$B\left(\omega_{1},t|r_{0}\right)=ir_{0}e^{-\alpha t}+\frac{k^{2}\omega_{1}}{2\alpha^{2}}\left(1-2e^{-\alpha t}+e^{-2\alpha t}\right)+im\left(1-e^{-\alpha t}\right),$$

and

(71) $$\begin{array}{ccc} C(\omega_{1},t|r_{0}) & = & i\omega_{1}r_{0}\frac{1}{\alpha }\left(1-e^{-\alpha t}\right)+\frac{k^{2}\omega_{1}^{2}}{2\alpha^{3}}\left[\alpha t-2\left(1-e^{-\alpha t}\right)+\frac{1}{2}\left(1-e^{-2\alpha t}\right)\right]\\ & & +im\omega_{1}\left[t-\frac{1}{\alpha }\left(1-e^{-\alpha t}\right)\right]. \end{array}$$

The characteristic function of the rate r(t) is obtained by setting $\omega_{2}=0$ in Equation (68), which also results in the Gaussian density

(72) $$\tilde{p}\left(\omega_{2},t|r_{0}\right)=\exp\left\{-\frac{k^{2}}{4\alpha }\left(1-e^{-2\alpha t}\right)\omega_{2}^{2}-i\left[r_{0}e^{-\alpha t}+m\left(1-e^{-\alpha t}\right)\right]\omega_{2}\right\},$$

and in the stationary state (t→∞), we have

(73) $${\tilde{p}}_{st}\left(\omega_{2}\right)=e^{-\left(k^{2}/4\alpha \right)\omega_{2}^{2}-im\omega_{2}}\;⟹\;p_{st}\left(r\right)={\left(\frac{\alpha }{\pi k^{2}}\right)}^{1/2}e^{-\alpha {\left(r-m\right)}^{2}/k^{2}},$$

which proves that the normal level *m* is the stationary mean value,

(74) m=E[r(t)].

It can also be shown that the correlation function of the process, defined as the average

$$C\left(\tau \right)=E\left[r(t+\tau )r(t)\right]-[E[r\left(t\right)]{]}^{2},$$

(τ≥0) in the stationary state reads [27]

(75) $$C\left(\tau \right)=\left(k^{2}/2\alpha \right)e^{-\alpha \tau },$$

which means that $\alpha^{-1}$ is the correlation time, $\tau_{c}$, of the rate. Indeed,

$$\tau_{c}\equiv \frac{1}{C(0)}\int_{0}^{\infty }C\left(\tau \right)d\tau =\alpha^{-1}.$$

Let us observe that the volatility, $\sigma^{2}=C\left(0\right)$, is independent of the normal level and given by

(76) $$\sigma^{2}=k^{2}/2\alpha .$$

The discount function $D(t|r_{0})$ is also obtained from Equations (68)–(71) although, in this case, after setting $\omega_{1}=-i$ and $\omega_{2}=0$ (cf. Equation (31)). We have

$$\begin{array}{ccc} \ln D(t) & = & -\frac{r_{0}}{\alpha }\left(1-e^{-\alpha t}\right)\\ & + & \frac{k^{2}}{2\alpha^{3}}\left[\alpha t-2\left(1-e^{-\alpha t}\right)+\frac{1}{2}\left(1-e^{-2\alpha t}\right)\right]-m\left[t-\frac{1}{\alpha }\left(1-e^{-\alpha t}\right)\right], \end{array}$$

which, after rearranging terms, can be written as

(77) $$\ln D\left(t\right)=-\left(m-\frac{k^{2}}{2\alpha^{2}}\right)t+\frac{1}{\alpha }\left[m-r_{0}-\frac{k^{2}}{4\alpha^{2}}\left(3-e^{-\alpha t}\right)\right]\left(1-e^{-\alpha t}\right),$$

where $r_{0}=r\left(0\right)$ is the initial rate. Note that, as t→∞ (in fact when $t\gg \alpha^{-1}$, i.e., for times much greater than the correlation time $\alpha^{-1}$) Equation (77) shows at once that the discount function of the Vasicek model has the typical exponential decay

(78) $$D\left(t\right)≃e^{-r_{\infty }t},$$

where

(79) $$r_{\infty }=m-k^{2}/2\alpha^{2},$$

is the long-run discount rate. Let us note that the long-run rate can be defined as the limit

(80) $$r_{\infty }=-\lim_{t\to \infty }\frac{\ln D(t)}{t},$$

as long as the limit exists. Let us also note the important fact that $r_{\infty }$ is smaller than the mean value of the return given by the normal level *m*. This reduction is quantified by the “noise-to-signal” ratio k/α, which means that either a long persistence (recall that this is equivalent to long correlation time, i.e., α small) or an increase of the noise fluctuations (i.e., *k* large) reduce the long-run discount rate as compared with the average rate *m*.

Finally, we easily see from Equation (77) that, as t→0, the discount function approximates to $D\left(t\right)≃e^{-r_{0}t}$, which would correspond to a fixed interest rate without random fluctuations or deterministic changes.

#### Risk Aversion

As mentioned above, risk aversion is taken into account by introducing the market price of risk q(r) and changing the drift according to Equation (32). For the Vasicek model, in which f(r)=−α(r−m) and g(r)=k, we have

(81) $$f^{*}\left(r\right)=-\alpha \left(r-m\right)+kq\left(r\right),$$

and assuming q(r)=q to be a constant independent of *r*, we write

(82) $$f^{*}\left(r\right)=-\alpha \left(r-m^{*}\right),$$

where

(83) $$m^{*}=m+\frac{qk}{\alpha }.$$

Since the modified drift $f^{*}\left(r\right)$ has the same form that f(r), we conclude that the adjusted-for-risk discount function will be given by Equation (77) after the replacement $m\to m^{*}$. In particular, the adjusted long-run discount now reads (cf. Equation (79))

(84) $$r_{\infty }^{*}=m+\frac{qk}{\alpha }-\frac{k^{2}}{2\alpha^{2}}.$$

We thus see that the long-run discount depends on the historical rate *m*; however, this is shifted by two terms. The first term raises the long-run rate due to the market price of risk. The second shift lowers it by an amount given by the ratio of uncertainty (as measured by *k*) and persistence (as measured by α). We rewrite Equation (84) as

(85) $$r_{\infty }^{*}=m+\frac{k}{\alpha }\left(q-\frac{k}{2\alpha }\right).$$

This shows that the overall shift in the long-run discount rate will be positive or negative depending on the size of the market price of risk and on the noise-to-signal ratio between the volatility parameter and the reversion rate.

It is not surprising that the market price of risk raises the long term rate; however, it is not so obvious that uncertainty and persistence can lower it. Indeed, for any given mean interest rate *m*, by varying *k* and α, the long-run discount rate $r_{\infty }$ can take on any value less than *m*, including negative values, while, at the same time, the standard deviation σ can also be made to take on any arbitrary positive value.

A negative long-run rate is due to the amplification of negative real interest rates r(t). Computation of the discount function involves an average over exponentials, rather than the exponential of an average. As a result, periods where interest rates are negative are amplified and can easily dominate periods where interest rates are large and positive, even if the negative rates are rarer and weaker. It does not take many such periods to substantially reduce the long run interest rate.

To summarize, in the Vasicek model, and even taking into account risk aversion, the long-run discounting rate can be much lower than the mean and, indeed, can correspond to low interest rates that are rarely observed.

### 4.3. The Cox–Ingersoll–Ross (Feller) Model

In the financial literature, one of the most accepted models for interest rates is the Cox–Ingersoll–Ross (CIR) model [42] where rates follow the Feller process described by drift and noise intensity given, respectively, by [45]

(86) $$f\left(r\right)=-\alpha \left(r-m\right),\;g\left(r\right)=k\sqrt{r}.$$

The Feller model is thus a diffusion process described by the stochastic differential equation

(87) $$dr\left(t\right)=-\alpha \left[r(t)-m\right]dt+k\sqrt{r(t})dW\left(t\right),$$

where W(t) is the standard Wiener process, and, as in the OU process, m>0 represents the mean stationary rate (the *normal level*), and $\alpha^{-1}$ is the correlation time [27]. Let us note that, in one-dimensional diffusions, the diffusion coefficient is given by the square of the noise intensity, and we thus see that the Feller process has a linear diffusion vanishing at the origin. This turns the origin into a singular boundary, which results in significant properties for the process [43].

As in the Vasicek model, the linear drift results in a restoring force, which, in the absence of noise, makes the process decay toward the normal level *m*. On the other hand, the state-dependent noise intensity $k\sqrt{r}$ for large values of *r* magnifies the effect of noise, while when *r* goes to zero, this effect vanishes. Therefore, as the process approaches the origin, the drift drags *r* towards *m*. Hence, since m>0, starting at some positive value $r_{0}>0$ the process cannot attain negative values with the overall result that *the Feller process always remains positive*.

Previous works [27,43] that we reviewed rather thoroughly presented the properties of the Feller process, and we refer the reader to these works for more detailed information. The process is not Gaussian, and the stationary PDF as t→∞ is the Gamma distribution [27]

(88) $$p_{st}\left(r\right)=\frac{{\left(2\alpha /k^{2}\right)}^{\theta }}{\Gamma (\theta )}r^{\theta -1}e^{-(2\alpha /k^{2})r},$$

where

(89) $$\theta =\frac{2\alpha m}{k^{2}}$$

is a positive and dimensionless constant that combines all the parameters of the model into a single expression. As mentioned above, a major characteristic of the Feller process is that r(t) cannot attain negative values, which makes the model a convenient tool for pricing bonds, which are never negative [13].

In the Feller model, the joint density of the discounting process (x(t),r(t)) defined in Equation (16) obeys the Fokker–Planck equation (FPE) (cf. Equations (17) and (18))

(90) $$\frac{\partial p}{\partial t}=-r\frac{\partial p}{\partial x}+\alpha \frac{\partial }{\partial r}\left[\left(r-m\right)p\right]+\frac{k^{2}}{2}\frac{\partial^{2}}{\partial r^{2}}\left(rp\right),$$

with the initial condition

(91) $$p\left(x,r,0|r_{0}\right)=\delta \left(x\right)\delta \left(r-r_{0}\right).$$

The joint Fourier transform, Equation (30), turns Equations (90)–(91) into a more manageable problem:

(92) $$\frac{\partial \tilde{p}}{\partial t}=\left(\omega_{1}-\alpha \omega_{2}-i\frac{k^{2}}{2}\omega_{2}^{2}\right)\frac{\partial \tilde{p}}{\partial {\tilde{\omega }}_{2}}-i\alpha m\omega_{2}\tilde{p},$$

(93) $$\tilde{p}\left(\omega_{1},\omega_{2},0|r_{0}\right)=e^{-i\omega_{2}r_{0}}.$$

Equation (92) is a linear partial differential equation of first order whose solution can be obtained by the method of characteristics, and we refer the interested reader to our work [27] for a detailed information. Once we know the solution $\tilde{p}\left(\omega_{1},\omega_{2},t|r_{0}\right)$, the discount function is then obtained through Equation (31) with the result [27]

(94) $$D\left(t\right)={\left[\frac{2\lambda e^{-(\lambda -\alpha )t/2}}{\left(\lambda +\alpha \right)+\left(\lambda -\alpha \right)e^{-\lambda t}}\right]}^{\theta }\exp\left\{-\frac{2\left(1-e^{-\lambda t}\right)r_{0}}{\left(\lambda +\alpha \right)+\left(\lambda -\alpha \right)e^{-\lambda t}}\right\},$$

where θ is defined in Equation (89) and

(95) $$\lambda =\sqrt{\alpha^{2}+2k^{2}}.$$

Notice that λ>α and the time scale represented by $\lambda^{-1}$ is smaller than the correlation time $\alpha^{-1}$.

In this case, the long-run discount rate, defined by the limit (cf. Equation (80))

$$r_{\infty }=-\lim_{t\to \infty }\frac{\ln D(t)}{t},$$

is directly obtained from Equation (94) with the result

(96) $$r_{\infty }=\frac{1}{2}\left(\lambda -\alpha \right)\theta ,$$

and, as in the Vasicek model, the effective discount reduces to the expected exponential decay

(97) $$D\left(t\right)≃e^{-r_{\infty }t}\;\;\left(t\to \infty \right).$$

Substituting into Equation (96) the expressions for θ and λ given in Equations (89) and (95), we write

(98) $$r_{\infty }=\frac{2m}{1+\sqrt{1+2k^{2}/\alpha^{2}}},$$

which clearly shows that the long-run discount rate is always smaller than the stationary average rate:

$$r_{\infty }<m.$$

**Figure 1:**
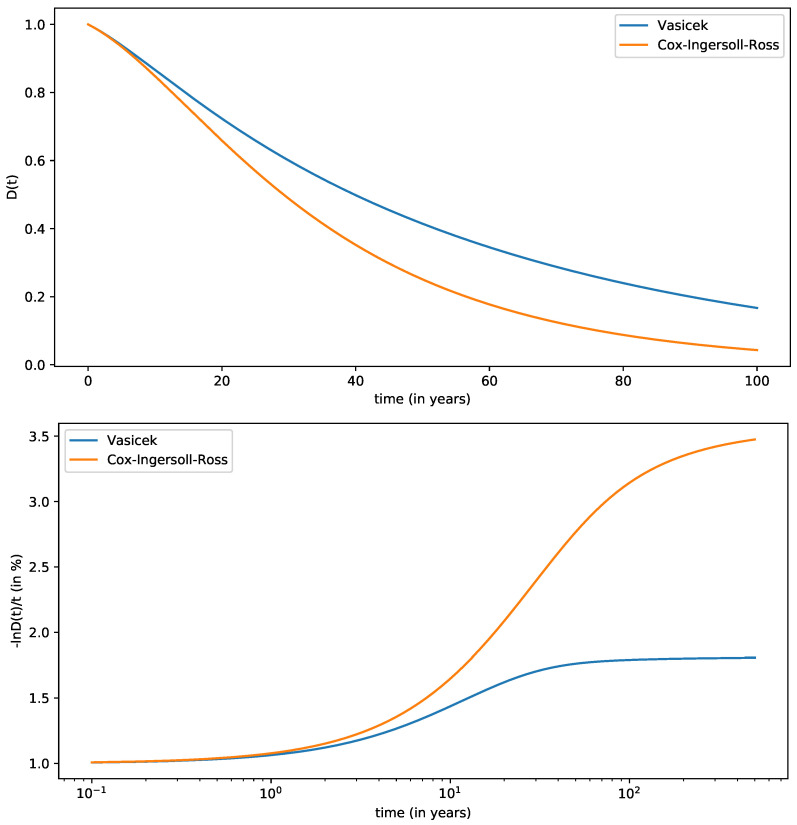
The Vasicek and Cox–Ingersoll–Ross discount functions. The parameters used are those corresponding to the United States and are provided by Table 5 of Ref. [29] (see Section 5). In the top figure, we plot the discount function D(t), while in the bottom figure, we plot the log ratio −lnD(t)/t. In the top figure, we observe the asymptotic exponential decay of the discount after more than a hundred years, while in the bottom figure, we clearly see the existence of a long-run discount rate for the Vasicek model (cf. Equation (80)). The initial rate $r_{0}$ is arbitrarily taken to be 1%. In both models, we assume no market price of risk q(r)=0 (the Local Expectation Hypothesis).

Figure 1 shows the discount function D(t) along with the quantity −lnD(t)/t (cf. Equation (80)) and compare them with the Vasicek model with equivalent parameters.

#### Risk Aversion

For the Feller process, the adjusted drift for risk defined in Equation (54) reads

(99) $$f^{*}\left(r\right)=-\alpha \left(r-m\right)+kq\left(r\right)\sqrt{r},$$

where q(r) is the market price of risk as discussed in the previous section. For any function q(r) (including a constant market price of risk *q*), this adjusted drift leads to an unsolvable Fokker–Planck equation with no analytical expression for the adjusted discount and the long-run discount rate. It is, nonetheless, possible to obtain analytical expressions for these quantities if the market price of risk has the following functional form

(100) $$q\left(r\right)=q\sqrt{r},$$

where q≥0 is a positive quantity. In such a case, we may write

(101) $$f^{*}\left(r\right)=-\alpha^{*}\left(r-m^{*}\right),$$

where

(102) $$\alpha^{*}=\alpha -kq,\;m^{*}=\frac{\alpha m}{\alpha -kq}.$$

The adjusted drift has the same form as f(r). Therefore, the adjusted discount function will be given Equation (94) with the replacements $\alpha \to \alpha^{*}$ and $m\to m^{*}$, and the long-run discount is (cf. Equation (98))

(103) $$r_{\infty }^{*}=\frac{2m^{*}}{1+\sqrt{1+2k^{2}/{\alpha^{*}}^{2}}}.$$

From the definitions of $\alpha^{*}$ and $m^{*}$, we easily see that $\alpha^{*}\leq \alpha$ and $\alpha^{*}m^{*}=\alpha m$. Hence, writing $r^{*}$ as

$$r_{\infty }^{*}=\frac{2\alpha^{*}m^{*}}{\alpha^{*}+\sqrt{{\alpha^{*}}^{2}+2k^{2}}}\geq \frac{2\alpha m}{\alpha +\sqrt{\alpha^{2}+2k^{2}}}=r_{\infty },$$

so that $r_{\infty }^{*}\geq r_{\infty }$, and, if the market price of risk has the form given in Equation (100), then, in the CIR model, risk always increases the long-run discount rate regardless of the noise intensity and persistence.

### 4.4. The Log-Normal Model

In this model, rates are described by the the geometric Brownian motion (log-normal process), and the model is determined by the stochastic differential equation

(104) $$\frac{dr}{r}=\alpha dt+kdW\left(t\right),$$

where *r* is the interest rate, α and *k* are constant parameters. α may be positive or negative, whereas *k* is always positive, and W(t) is the standard Wiener process. Equation (104) can be integrated at once yielding

(105) $$r\left(t\right)=r_{0}\exp\left\{\left(\alpha -\frac{k^{2}}{2}\right)t+kW\left(t\right)\right\},$$

showing that r(t) is never negative ($r_{0}>0$). Therefore, the log-normal model is more suited for modeling nominal interest rates and bonds in finance than for the long-run real rates of environmental economics. Contrary to the OU and Feller processes, the log-normal process does not show reversion to the mean. Indeed, as *t* increases, we see from Equation (105) that the rate either diverges when α>0 or goes to zero if α<0. In an equivalent way, one can also show from Equation (105) that the mean and variance of the process are [27]

$$\langle r\left(t\right)\rangle =r_{0}e^{\alpha t},\;\mathrm{Var}\left[r\left(t\right)\right]=r_{0}^{2}e^{2\alpha t}\left(e^{k^{2}t}-1\right).$$

The discount associated with the log-normal process model was studied in 1978 by L. U. Dothan [44], and, in finance, it is sometimes refereed to as the Dothan model. As it allows for analytical treatment, it is one of the models used in the literature [13]. For this model, the FPE for the joint density of the discounting processes (x(t),r(t)) is given by (cf. Equation (17))

(106) $$\frac{\partial p}{\partial t}=-r\frac{\partial p}{\partial x}-\alpha \frac{\partial }{\partial r}\left(rp\right)+\frac{1}{2}k^{2}\frac{\partial^{2}}{\partial r^{2}}\left(r^{2}p\right),$$

with the usual initial condition given by Equation (18). The Fourier transform of this expression leads to the following equation for the characteristic function $\tilde{p}\left(\omega_{1},\omega_{2},t|r_{0}\right)$

(107) $$\frac{\partial \tilde{p}}{\partial t}=\left(\omega_{1}+\alpha \omega_{2}\right)\frac{\partial \tilde{p}}{\partial {\tilde{\omega }}_{2}}+\frac{1}{2}k^{2}\omega_{2}^{2}\frac{\partial^{2}\tilde{p}}{\partial {\tilde{\omega }}_{2}^{2}}$$

and the initial condition (91). Equation (106) is a partial differential equation of second order, which cannot be solved by the method of characteristics, and we refer the interested reader to our work [27] for more information on how to solve Equation (107) using the time–Laplace transform. Hence—and contrary to Vasicek and CIR models where it is possible to obtain exact expressions for the discount function D(t)—for the log-normal case, we can only achieve the exact expression of its Laplace transform,

$$\hat{D}\left(s\right)=\int_{0}^{\infty }e^{-st}D\left(t\right)dt.$$

The resulting formula—written as an integral of special functions, the Kummer function—is rather intricate, and we will not write it here (see [27] for more information). However, from the exact expression for $\hat{D}\left(s\right)$, we can obtain asymptotic expressions as t→∞ of the discount function D(t) in real time. This is done using the so-called Tauberian theorems, which relate the small *s* behavior of $\hat{D}\left(s\right)$ with the long-time behavior of D(t) [46,47]. The final result is the following asymptotic expression for the discount function D(t) in the long run as t→∞ [27]

(108) $$D\left(t\right)∼\left\{\begin{array}{cc} \mathrm{constant} & \;k^{2}/2>\alpha ,\\ e^{-r_{\infty }t} & \;k^{2}/2<\alpha ,\\ t^{-1/2} & \;k^{2}/2=\alpha . \end{array}\right.$$

The asymptotic form of the discount function thus depends on the values taken by the ratio $\alpha /k^{2}$ between the strength of the constant deterministic drift α and the amplitude of fluctuations given by $k^{2}/2$ (which can be considered the “signal-to-noise ratio” of this model).

(i) The case $k^{2}/2>\alpha$ corresponds to strong fluctuations, where the noise intensity $k^{2}/2$ is greater than the drift parameter α. In this case, the discount tends to a constant value (for the actual value of this constant, see [27]).

(ii) The case $k^{2}/2<\alpha$ corresponds to mild fluctuations for which the deterministic drift is stronger than noise. In such a case, the discount function has the expected exponential decay

(109) $$D\left(t\right)∼e^{-r_{\infty }t},$$

with a long-run rate of discount given by [27]

(110) $$r_{\infty }=\frac{1}{\delta }\left(\alpha -\frac{k^{2}}{2}\right),$$

where δ>1 is a positive numerical factor that only depends on the ratio $2\alpha /k^{2}$ and reads

(111) $$\delta =\psi \left(2\alpha /k^{2}\right)+\frac{1}{2\alpha /k^{2}-1},$$

where ψ(·) is the digamma function.

Let us write Equation (109) in a more characteristic form. Indeed, from Equation (105), we see that

$$E\left[\ln\frac{r(t)}{r_{0}}\right]=\left(\alpha -\frac{k^{2}}{2}\right)t,$$

and, with the help of Equation (109), we write Equation (109) as

(112) $$D\left(t\right)∼\exp\left\{-\frac{1}{\delta }E\left[\ln\frac{r(t)}{r_{0}}\right]\right\},$$

(t→∞ and $k^{2}/2<\alpha$). Note that the average $E[\ln r\left(t\right)/r_{0}]$ is what a practitioner would take as an estimate of the discount rate up to time *t* within the log-normal model. Since δ>1, the analytical result (112) shows that the actual long-run rate of the model is a fraction of the average rate. We indicated elsewhere that the long-run discount rate is at most 73% of the average rate [26].

In this way, when $2\alpha /k^{2}>1$, the log-normal model follows a similar pattern to that of the OU and Feller models: In all of them, the long-run rate is smaller than the average rate. This general statement is a direct consequence of Jensen’s inequality, which states that the average of a convex function is greater than or equal to the function of the average; that is, E[f(X)]≥f(E[X]). Assuming *f* to be the decreasing exponential and *X* as the cumulative process x(t) defined in Equation (14), it follows immediately that the long-run rate $r_{\infty }$ must be always less than or equal to the average rate. Nonetheless, our procedure quantifies the difference among averages [27].

(iii) The critical case $\alpha =k^{2}/2$, in which deterministic motion and fluctuations are balanced, leads to the hyperbolic discount function as obtained by Farmer and Geanakoplos [48,49]. The hyperbolic D(t) is substantially greater than any exponential decaying function, showing that there is no long-run rate of interest in this case. In fact, the long-run rate of interest is 0; however, that does not convey as precise information as saying that D(t) is approximately $k/\sqrt{t}$ for all large *t*. Since the sum (i.e., the integral) of all these D(t) is infinite, such D(t) assigns infinite value to any permanent positive flow of consumption: the infinite future is infinitely valuable.

#### Risk Aversion

Let us very briefly comment on the inclusion of risk aversion in the Dothan model. For the log-normal process f(r)=αr and g(r)=kr and

$$f^{*}\left(r\right)=\left[\alpha +kq\left(r\right)\right]r.$$

Assuming a constant market price of risk, q(r)=q≥0, we have

$$f^{*}\left(r\right)=\alpha^{*}r,\;\alpha^{*}=\alpha +q.$$

Again, $f^{*}\left(r\right)$ has the same form than f(r), and all previous results will apply after making the replacement α→α+q.

## 5. Some Empirical Results

In order to choose an appropriate model for rates that would allow us to obtain realistic long-run discount functions, we performed a rather complete empirical study on interest rates combined with inflation. Our study follows the line partly initiated by Newell and Pizer [50] (see also [51]). To our knowledge, there are few empirical studies on real rates with some exceptions. We remark here the recent and excellent survey by Giglio et al. on the housing market in London and Singapore [52,53,54], which allowed for a rather realistic estimation of long-run discount rates.

Our first concern was knowing how the discount process depended on the underlying random process that characterizes interest rates. To this end, we collected data for the nominal interest rates and inflation of fourteen countries over time spans ranging from 87 to 318 years [26]. The countries in our sample are Argentina (ARG, 1864–1960), Australia (AUS, 1861–2012), Chile (CHL, 1925–2012), Germany (DEU, 1820–2012), Denmark (DNK, 1821–2012), Spain (ESP, 1821–2012), United Kingdom (GBR, 1694–2012), Italy (ITA, 1861–2012), Japan (JPN, 1921–2012), Netherlands (NLD, 1813–2012), Sweden (SWE, 1868–2012), the United States (USA, 1820–2012) and South Africa (ZAF, 1920–2012). The data are summarized in Table 2.

**Figure 2:**
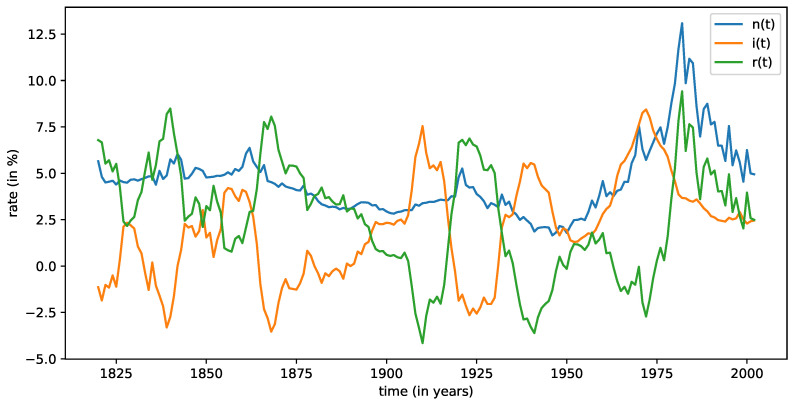
The construction of real interest rates r(t) in terms of the nominal rates n(t) and inflation i(t) (Fisher’s procedure). Large fluctuations and negative rates are shown here for the United States (USA).

Since all but two of our nominal interest rate processes are for 10-year government bonds, which pay out over a 10-year period, we smoothed out inflation rates with a 10-year moving average and subtracted the annualized inflation index from the annualized nominal rate to compute the real interest rate, as explained in the previous section by means of the Fisher’s procedure (cf. Equation (61)),

r(t)=n(t)−i(t),

where n(t) is the nominal rate and i(t) is the inflation rate. The particular case of the United States is plotted in Figure 2.

In our empirical analysis, the nominal rates are determined by IG rates constructed from the 10-year Government Bond Yield y(t,τ) with τ= 10 years. Thus, looking at Equations (63) and (64), we estimate the nominal rates by

n(t)∼y(t,τ=10 years).

Let us recall that denoting, by B(t,t+τ), the government bond issued at time *t* and maturing at time t+τ with unit maturity, B(t,t)=1, the yield y(t,τ) is defined as (cf. Equation (38))

$$y\left(t,\tau \right)\equiv -\frac{1}{\tau }\ln B\left(t,t+\tau \right)\;⟹\;B\left(t,t+\tau \right)=e^{-\tau y(t|\tau )}.$$

One can argue that τ=10 years is not a short period of time in order to consider y(t,τ=10 years) a very accurate estimator of n(t) (cf. Equations (63) and (64)). Although this may be true, we must bear in mind that 10 year bonds are the shortest bonds available for most of the countries analyzed.

The inflation rate is estimated through the Consumer Price Index (CPI) as

$$i\left(t\right)∼\frac{1}{\tau }\ln[I\left(t+\tau \right)/I\left(t\right)],$$

where I(t) is the aggregated inflation up to time *t*, and τ=10 years. The relation between I(t) and the Consumer Price Index (CPI) is

$$I\left(t+\tau \right)=I\left(t\right)\prod_{j=0}^{\tau -1}\left[1+C(t+j)\right],$$

where C(t) is the time series of the empirical CPI. The instantaneous rate of inflation i(t) is, therefore, estimated by the quantity i(t+τ), which is written in terms of the CPI reads

$$i\left(t\right)∼i\left(t+\tau \right)=\frac{1}{\tau }\sum_{j=0}^{\tau -1}\ln\left[1+C(t+j)\right].$$

A remarkable characteristic observed for all countries is that real interest rates frequently become negative as the real interest rates are mostly dominated by inflation i(t)>0 (see Figure 2). In some cases, as we can see in Table 3 (see also Figure 2), negative real rates show high frequency and long periods of time, and, on average, real interest rates are negative one quarter of the time.

This makes the Feller and log-normal models—as well as any other model assuming positive interest rates [13]—less interesting or at least less appropriate to model real interest r(t)=n(t)−i(t) instead of solely considering nominal rates n(t). It is, however, necessary to remember the fact that nominal rates can indeed become negative as has recently been observed in Western economies. We, therefore, confined the empirical work to the OU (Vasicek) model and then assumed the Local Expectation Hypothesis [36,37,38], according to which, we live in a risk neutral world, and the market price of risk is zero. Let us recall, as explained in Section 3, that the market price of risk q=q(r,t) may be any function of the rate and time. There is, hence, no unique expression for it. Thus, in Section 4, we presented several expressions of the long-run rate, which include risk in different forms for all market models analyzed. The usual assumption in the literature [33,38] is that the price of risk is a constant that is independent of time and the value of the rate but without any empirical justification. This is a sensitive issue since data is quite scarce, particularly in environmental applications, for obtaining a credible estimation of *q*. Moreover, to our knowledge, in environmental problems, the estimations of the long-run rates do not take into account, nor even mention, the market price of risk [14,15,16,50,54]. In any case, we do not lessen the importance of taking into account some kind of risk in estimating log-run rates; however, unfortunately, with the data available to us, we cannot make any reliable estimation of *q*. For this reason, we have not taken into account the market price of risk, assuming risk-neutral investors and following the Local Expectation Hypothesis. In any case, the question is under consideration).

We can estimate the parameters *m*, α and *k* of the Vasicek model to each of the data series. There are several possible procedures. One of the possible methods is to deal with stationary averages. The parameter *m* can be estimated through the stationary mean value of the rate (cf. Equation (74))

m=E[r(t)].

Parameters α and *k* can be estimated via the correlation function of the Ornstein–Uhlenbeck process. Thus, from Equation (75), we have

$$C\left(t-t^{\prime }\right)=\frac{k^{2}}{2\alpha }e^{-\alpha |t-t^{\prime }|}.$$

The empirical correlation can then be fitted by an exponential, which in turn allows us to estimate α (measured in 1/year units) for each country. The parameter *k* is obtained from the empirical standard deviation $\sigma^{2}={E[|r\left(t\right)-m|}^{2}]$, and for the Vasicek model, it is given by

$$k=\sigma \sqrt{2\alpha }.$$

The resulting parameters are shown in Table 3. The minimum and maximum values for each country allows us to show that parameters may indeed fluctuate over different periods of time.

Finally, the long-run discount rate can be evaluated from Equation (79),

$$r_{\infty }=m-k^{2}/2\alpha^{2}.$$

For this calculation, we neglected the market price of risk as mentioned above.

The countries studied can be divided into two groups. Nine countries have long-run positive rates (boldface in Table 3). The average historical rate for these nine countries is $\bar{m}=2.7$% while the average long-run rate is ${\bar{r}}_{\infty }=2.1$%, which, on average, is 29% lower than $\bar{m}$. Five countries with less stable behavior have long-run negative rates and an exponentially increasing discount.

Four cases of this group have a negative average rate *m* due to at least one period of runaway inflation; the exception is Spain, which has a (highly positive) mean real interest rate but still has a long-run negative rate. Convergence in this case to the long-run rate happens within 30 years and typically within less than a decade. This contrasts with other treatments, which assume that short term rates are always (or nearly always) positive and predict that the decrease in the discounting rate happens over a much longer timescale, which can be measured in hundreds of years [50,51,55,56,57,58].

Alternatively, we can estimate parameters using the well-established maximum likelihood procedure. For the Vasicek model, the maximum likelihood estimation is extensively documented in the financial mathematics literature (see for instance [13]). The approach differs from the previous one as it focuses attention on two consecutive steps of our time series (generally consecutive years) and takes the conditional probability to perform the estimation. Table 4 shows that the most inaccurate estimator is $\hat{\alpha }$, an unsurprising fact since the estimation of α is known to be quite difficult for the Vasicek model [59]. The last two columns in Table 4 include the long-run interest rate estimator ${\hat{r}}_{\infty }$ and its error calculated through error propagation.

Only four countries (the Netherlands, Sweden, the United Kingdom and the United States) show a positive long-run rate, $r_{\infty }>0$. This estimation procedure leads to more negative $r_{\infty }$. This feature can be attributed to the fact that, in most of the countries, estimating α via the maximum likelihood brings smaller values, which in turn leads to more drastic corrections to the long-run rate as $r_{\infty }$ is inversely proportional to α (remember that $r_{\infty }=m-k^{2}/2\alpha^{2}$). This effect is particularly relevant in most turbulent countries during last century (e.g., Germany) thus signaling a more intense lack of stationarity in empirical data. The averaged $r_{\infty }$ over all countries estimated via maximum likelihood is also sensitively smaller.

However, if we focus the attention on stable countries (with $r_{\infty }>0$) both estimation procedures bring quite similar results (see, for instance, the United States case in Table 3 and Table 4, 2.1% versus 1.8%). As in the previous estimation procedure, we also neglected the effects of risk aversion and the market price of risk.

The Vasicek model is therefore the only one among the three classic models allowing for negative rates, and for this reason, both the Feller and the log-normal models have been excluded from our analysis. However, for the Cox–Ingersoll–Ross (Feller) model, it is possible to redefine the model by shifting the process $y=r-r_{min}$ where $r_{min}<0$.

The estimation through the maximum likelihood procedure and its error analysis is then possible [59], and Figure 1 includes the shifted Cox–Ingersoll–Ross discount and compares it with the equivalent result assuming the Vasicek model. We demonstrated in Ref. [29] how to redefine the Feller process and how maximum likelihood estimation could be possible.

## 6. Discussion

We reviewed one of the most important aspects of economics and finance, i.e., the problem of discount, which weights the future relative to the present. The problem is clearly very relevant in finance over relatively short time spans; however, it is even more crucial for long-run planning in addressing environmental problems on how to act now with measures to mitigate the effects of climate change.

To our knowledge, this is a rather unknown issue to the econophysics community, and this review is particularly intended for this group. We thus addressed the problem with a simple approach and yet with a high level of rigor and generality. In this way, we also developed the traditional method used in mathematical finance to address the problem, i.e., the Feynman–Kac approach. In addition, we reviewed the bond pricing theory and its close similarity with discounting and presented a short introduction to the term structure of interest rates along with the market price of risk.

We obtained quantitative results on the problem by studying, in some detail, three standard models for the dynamical evolution of rates. These models are based on the Ornstein–Uhlenbeck process (the Vasicek model), thus, allowing for both positive and negative rates and also on the Feller and log-normal processes for positive rates. We presented the exact results for the discount function and asymptotic expressions as t→∞ leading to the long-run discount rate, and we discussed the modifications of these expressions when the market price of risk is taken into account.

An important conclusion is that, for all models, the long-run discount rate is always less than the long-time average rate. This is a conclusion that necessarily has to have consequences in any long-run economic planning. Finally, we reviewed our recent empirical study on 14 different countries, which obtained numerical values for the parameters that appear in the Vasicek model. We demonstrated two different estimation procedures and briefly discussed their differences and similarities.

## Figures and Tables

**Table 1:** Key statistical features for three standard models: the Vasicek (Ornstein–Uhlenbeck), the Cox–Ingersoll–Ross (Feller) and the log-normal models. The average and variance are provided in terms of the model parameters to better compare the asymptotic behavior of D(t). The asymptotic discount is provided by showing an exponential decay with a long-run rate of discount $r_{\infty }$ for the Vasicek and the Cox–Ingersoll–Ross models and also in the log-normal case for a specific combination of parameters ($k^{2}/2<\alpha$, mild fluctuations). The parameter δ is defined in Equation (111).

Model	E[r(t)]	Var[r(t)]	D(t→∞)		$r_{\infty }$
Vasicek	*m*	$k^{2}/\alpha$	$\exp(-r_{\infty }t)$		$m-k^{2}/2\alpha^{2}$
Feller	*m*	$mk^{2}/\left(2\alpha \right)$	$\exp(-r_{\infty }t)$		$\frac{2m}{1+\sqrt{1+2k^{2}/\alpha }}$
Log-normal	$r_{0}e^{\alpha t}$	$r_{0}^{2}e^{2\alpha t}\left[e^{k^{2}t}-1\right]$	constant	($k^{2}/2>\alpha$)	−−
			$\exp(-r_{\infty }t)$	($k^{2}/2<\alpha$)	$(\alpha -k^{2}/2)/\delta$
			$t^{-1/2}$	($k^{2}/2=\alpha$)	−−

**Table 2:** List of the countries analyzed. CPI stand for Consumer Price Index. Data has different specificities, particularly in terms of empty records as has been reported elsewhere [26,29]. *, We have taken the discount (ID) rate since the government bond yield data was not available.

Country	CPI	Bond Yield	From	To	Records
Italy	CPITAM	IGITA10	12/31/1861	09/30/2012	565
		annual from 12/31/1861	quarterly		
		quarterly from 12/31/1919			
Chile	CPCHLM	IDCHLM	03/31/1925	09/30/2012	312
		quarterly	quarterly		
Canada	CPCANM	IGCAN10	12/31/1913	09/30/2012	357
		quarterly	quarterly		
Germany	CPDEUM	IGDEU10	12/31/1820	09/30/2012	729
		annual from 12/31/1820	quarterly		
		quarterly from 12/31/1869			
Spain	CPESPM	IGESP10	12/31/1821	09/30/2012	709
		annual from 12/31/1821	quarterly		
		quarterly from 12/31/1920			
Argentina	CPARGM	IGARGM	12/31/1864	03/31/1960	342
		annual from 12/31/1864	quarterly		
		quarterly from 12/31/1932			
Netherlands	CPNLDM	IGNLD10D	12/31/1813	12/31/2012	189
		annual	annual		
Japan	CPJPNM	IGJPN10D	12/31/1921	12/31/2012	325
		quarterly	quarterly		
Australia	CPAUSM	IGAUS10	12/31/1861	09/30/2012	564
		annual from 12/31/1861	quarterly		
		quarterly 12/31/1991			
Denmark	CPDNKM	IGDNK10	12/31/1821	09/30/2012	725
		annual from 12/31/1821	quarterly		
		quarterly from 12/31/1914			
South Africa	CPZAFM	IGZAF10	12/31/1920	09/30/2012	329
		quarterly	quarterly		
Sweden	CPSWEM	IGSWE10	12/31/1868	09/30/2012	135
		annual	annual		
United Kingdom	CPGBRM	IDGBRD ${}^{*}$	12/31/1694	12/31/2012	309
		annual	annual		
United States	CPUSAM	TRUSG10M	12/31/1820	10/30/2012	183
		annual	annual		

**Table 3:** The OU (Vasicek) model parameter estimation in yearly units using stationary averages. “Neg RI” provides the time percentage and the number of years with negative real interest rates. The columns $\hat{m}$, $\hat{k}$ (in %) and $\hat{\alpha }$ are estimates from the country time series; ${\hat{r}}_{\infty }$ (in %) is evaluated from Equation (79). The Min and Max columns give reasons regarding the level of robustness of the estimation as they provide the minimum and the maximum values of the parameter estimation for four data blocks of equal length. The parameter α is estimated by fitting the empirical correlation function to an exponential (cf. Equation (75)) after using the whole data block. Countries in boldface are those considered historically more stable with positive long-run rates ${\hat{r}}_{\infty }>0$.

Country	Neg RI	$\hat{m}$	Min	Max	$\hat{k}$	Min	Max	$\hat{\alpha }$	${\hat{r}}_{\infty }$
Italy	28%(40y)	−0.3	−9.1	5.6	6.9	0.8	10.1	0.22	−5.4
Chile	56%(43y)	−6.8	−20.2	12.0	25.2	5.6	44.1	0.40	−26
**Canada**	22%(20y)	2.9	0.1	6	2.3	1.1	2.0	0.26	2.5
Germany	14%(25y)	−10.7	−51.0	4.0	33.9	0.9	61.4	0.20	−160
Spain	25%(45y)	5.7	−0.5	13.5	2.9	1.2	3.6	0.06	−6.4
**Argentina**	20%(17y)	2.4	−2.9	6.8	6.2	2.8	6.7	0.39	1.1
**Netherlands**	17%(33y)	3.2	0.8	5.4	1.6	0.8	2.2	0.14	2.4
Japan	33%(26y)	−2.2	−7.8	4.0	9.7	1.1	13.2	0.24	−10
**Australia**	23%(33y)	2.6	−0.7	4.9	2.3	0.7	2.8	0.19	1.9
**Denmark**	18%(33y)	3.2	1.5	4.3	2.3	1.1	2.9	0.23	2.7
**South Africa**	43%(36y)	1.8	−2.2	5.5	2.5	1.2	2.0	0.21	1.1
**Sweden**	28%(38y)	2.3	−0.3	3.9	2.5	0.6	3.4	0.25	1.9
**United Kingdom**	14%(45y)	3.3	1.4	4.3	1.9	1.0	2.4	0.19	2.8
**United States**	31%(36y)	2.6	1.0	4.0	1.8	1.2	2.1	0.18	2.1
**Stable countries**	23%(33y)	2.7	−0.14	5.0	2.6	1.04	2.94	0.23	2.1
Unstable counntries	31%(36y)	−2.9	17.7	1.8	16	1.9	26.5	0.22	−42

**Table 4:** Maximum likelihood estimation of the long-run interest rate for the Vasicek model. $\hat{m}$ estimates of the mean real interest rate in 1/years (in %). $\hat{\alpha }$ estimates the characteristic reversion time in 1/years. The squared root of $\hat{k^{2}}$ is given in terms of $1/{\left(\mathrm{year}\right)}^{3}$ (multiplied by $10^{4}$ to be comparable with the results in Table 3). These estimators are accompanied by the square root of the variance of each estimator. ${\hat{r}}_{\infty }$ estimates the long-run real interest rate with 1/year (in %). Negative values of ${\hat{r}}_{\infty }$ imply that the discount function is asymptotically increasing. The standard error is obtained through error propagation. The last two rows show the average over all countries with the more stable countries ($r_{\infty }>0$) and the less stable countries ($r_{\infty }<0$). The error provided corresponds to the standard deviation of the ${\hat{r}}_{\infty }$ for the different countries.

Country	$\hat{m}$	$\sigma_{\hat{m}}$	$\hat{\alpha }$	$\sigma_{\hat{\alpha }}$	$\hat{k^{2}}$	$\sigma_{\hat{k^{2}}}$	${\hat{r}}_{\infty }$	$\sigma_{{\hat{r}}_{\infty }}$
Italy	1.97	15.95	0.0056	0.0089	0.1146	0.068	−177.8	19.2
Chile	−5.79	31.46	0.0201	0.0227	31.07	2.49	−391.7	44.2
Canada	2.66	3.91	0.0142	0.0178	0.275	0.021	−4.15	3.94
Germany	−9.45	66.95	0.0071	0.0089	41.72	2.19	−4094	228
Spain	6.71	6.92	0.0167	0.0137	2.371	0.126	−35.78	7.28
Argentina	3.15	7.09	0.0228	0.0231	2.240	0.171	−18.31	7.27
**Netherlands**	**5.99**	0.78	0.1648	0.0550	**1.797**	0.243	**5.66**	0.78
Japan	5.02	24.68	0.0053	0.0114	1.396	0.109	−243.1	31.4
Australia	3.97	4.50	0.0089	0.0112	0.223	0.013	−10.29	4.58
South Africa	2.69	4.72	0.0154	0.0193	0.435	0.034	−6.49	4.77
**Sweden**	**2.79**	1.66	0.0676	0.0317	1.692	0.206	**0.95**	1.67
Denmark	4.10	2.59	0.0161	0.0133	0.315	0.017	−1.97	2.61
**United Kingdom**	**3.42**	0.62	0.1635	0.0326	**3.137**	0.253	**2.83**	0.62
**United States**	**3.19**	1.23	0.0603	0.0257	**1.003**	0.105	**1.81**	1.24
**Stable countries**	**3.85**	1.07	0.1140	0.0362	**1.907**	0.202	**2.81**	1.08
Unstable countries	1.50	16.86	0.0132	0.0150	8.120	0.523	−498.4	35.3

## Data Availability

The study reports data described and analyzed in Refs. [26,27,28,29,30].
